# Lectures on the Principles and Practice of Physic

**Published:** 1858-11

**Authors:** 


ART. VII.—*Lectures on the Principles and Practice of Physic; delivered at King's College, London.* By THOMAS WATSON, M. D., Fellow of the Royal College of Physicians; late Physician to the Middlesex Hospital, and formerly Fellow of St. John's College, Cambridge. A new American from the last Revised and Enlarged English Edition. With Additions, by Dr. FRANCIS CONDIE, M. D., Fellow of the College of Physicians of Philadelphia; Member of the American Philosophical Society, etc. etc. With one hundred and eighty-five Illustrations on Wood. Philadelphia: BLANCHARD & LEA. 1858.

We have just received from the publishers, the American reprint of the last English edition of "Watson's Practice." The English edition has just appeared, and has been hailed by the profession almost with enthusiasm.

We must certainly commend the enterprise of Messrs. Blanchard & Lea in being thus prompt in issuing the American reprint. This volume has been only lately received, but we notice it in advance of some which have been for some time on our table, on account of the opening of the sessions in Medical Schools. The importance to the student of a reliable and comprehensive work on Practice cannot be too highly estimated, and the progress of medical science is such that a work a few years old is necessarily imperfect in many respects. The lectures of Dr. Watson have been thoroughly revised and brought up to the present time. The additions are so extensive indeed, that in spite of a considerable enlargement in the size of the page, about two hundred pages of new matter have been added. The clearness and beauty of style which pervade this work, no less than the soundness of its teachings, have made it the most desirable text-book in the language; we do violence to our feelings of national pride in saying this, but it is our conviction, that, as yet, our country has produced no work on the Practice of Medicine equal to the one before us. The American editor has added a number of illustrations to this edition, with which long acquaintance has made us tolerably famliiar. These may be of some value, but we must own that we are weary of seeing the same cuts turning up in every available place, in the publications of this country. The mechanical execution, however, is excellent, and the new edition is indispensable to the library of every physician.

